# Maternal blood transcriptome as a sensor of fetal organ maturation at the end of organogenesis in cattle^†^

**DOI:** 10.1093/biolre/ioad103

**Published:** 2023-09-02

**Authors:** Maria Belen Rabaglino, José María Sánchez, Michael McDonald, Elena O’Callaghan, Pat Lonergan

**Affiliations:** School of Agriculture and Food Science, University College Dublin, Belfield, Dublin 4, Ireland; Departamento de Reproducción Animal, Instituto Nacional de Investigación y Tecnología Agraria y Alimentaria, Madrid, Spain; School of Agriculture and Food Science, University College Dublin, Belfield, Dublin 4, Ireland; School of Agriculture and Food Science, University College Dublin, Belfield, Dublin 4, Ireland; School of Agriculture and Food Science, University College Dublin, Belfield, Dublin 4, Ireland

**Keywords:** cattle, gestation, machine learning, transcriptomics, organ development

## Abstract

Harnessing information from the maternal blood to predict fetal growth is attractive yet scarcely explored in livestock. The objectives were to determine the transcriptomic modifications in maternal blood and fetal liver, gonads, and heart according to fetal weight and to model a molecular signature based on the fetal organs allowing the prediction of fetal weight from the maternal blood transcriptome in cattle. In addition to a contemporaneous maternal blood sample, organ samples were collected from 10 male fetuses at 42 days of gestation for RNA-sequencing. Fetal weight ranged from 1.25 to 1.69 g (mean = 1.44 ± 0.15 g). Clustering data analysis revealed clusters of co-expressed genes positively correlated with fetal weight and enriching ontological terms biologically relevant for the organ. For the heart, the 1346 co-expressed genes were involved in energy generation and protein synthesis. For the gonads, the 1042 co-expressed genes enriched seminiferous tubule development. The 459 co-expressed genes identified in the liver were associated with lipid synthesis and metabolism. Finally, the cluster of 571 co-expressed genes determined in maternal blood enriched oxidative phosphorylation and thermogenesis. Next, data from the fetal organs were used to train a regression model of fetal weight, which was predicted with the maternal blood data. The best prediction was achieved when the model was trained with 35 co-expressed genes overlapping between heart and maternal blood (root-mean-square error = 0.04, *R*^2^ = 0.93). In conclusion, linking transcriptomic information from maternal blood with that from the fetal heart unveiled maternal blood as a predictor of fetal development.

## Teaser

Co-expressed genes positively correlated with fetal weight at 42 days were biologically relevant for the organ. The maternal blood transcriptome accurately predicted fetal weight after modeling with the fetal heart data.

## Graphical Abstract

**Graphical Abstract:**
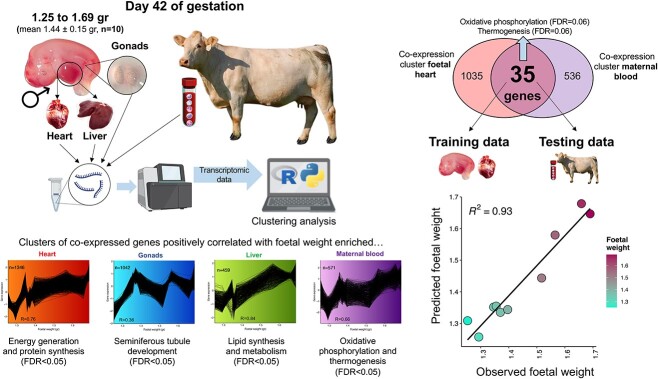

## Introduction

The embryonic and fetal periods are those imposing the most pronounced changes in the life of an individual. In mammals, pregnancy strongly alters maternal physiology, leading to metabolic and endocrine adaptations. In humans, given the invasive nature of placentation, circulating cell-free fetal DNA and RNA are detectable in maternal blood and can be measured to track pregnancy progression accurately and to diagnose pregnancy-associated pathologies [1–4]. In species with more superficial placentation, the use of maternal blood for pregnancy-related diagnoses is more limited. Nonetheless, in the cow, measurement of circulating pregnancy-associated glycoproteins, synthesized by the giant binucleate cells of the developing trophoblast that migrate to the endometrial epithelium initiating conceptus attachment [5], has been used to successfully determine pregnancy status [6, 7] and more recently, to determine the timing of presumptive conceptus attachment [8, 9]. Furthermore, expression of interferon-stimulating genes in circulating maternal blood leukocytes is used as an early indication of pregnancy [10]. However, changes in maternal blood reflecting fetal growth and organ maturation are still unknown for cattle.

To explore this aspect, a model is necessary where fetal growth can be stimulated non-pharmacologically. Previous studies in cattle demonstrated that progesterone (P4) concentrations during the first week post-ovulation directly influence the endometrial transcriptome [11] which, in turn, impacts embryonic trophoblast elongation [12]. High P4 concentrations advance the temporal changes that occur in the uterus, resulting in longer conceptuses [12–14] which produce more interferon-tau [14], the maternal recognition of pregnancy signal in ruminants [15]. Based on this knowledge, we used a previously established asynchronous embryo transfer model, known to accelerate conceptus elongation [16, 17] in association with an advanced uterine environment [18], to demonstrate that transferring Day 7 blastocysts into a Day 9 uterus, i.e., primed with higher P4 concentrations, led to heavier and larger Day 42 fetuses [19]. That study [19] generated fetuses with different weights at 42 days of gestation, recognized as the beginning of the fetal period in cattle [20]. Here, we studied 10 of those fetuses (all males) to test the hypothesis that the observed variations in fetal growth also impacted the maturation of organs differentiated by this stage, such as the liver, gonads, and heart. We tested this hypothesis through a transcriptomic approach since gene expression coordinates embryonic development and organ function [21]. Furthermore, somatic organs undergo a main period of transcriptional change during the embryonic/fetal period, which is characterized by an increase in the expression of genes with early organ-specific functions. The human brain, liver, and gonads experience this period around 5–6 weeks after conception or at around 4 weeks for the heart [22]. As the heart becomes functional earlier than the other organs [23], and its development is closely related to that of the placenta [24, 25], we hypothesize that molecular changes in the heart can potentially influence the maternal blood transcriptome. The objectives of this study were to determine the transcriptomic modifications in maternal blood and fetal liver, gonads (testes), and heart according to fetal weight and to model a molecular signature based on the fetal organs allowing the prediction of fetal weight from the maternal blood transcriptome.

Functionally related genes are often co-regulated [26, 27]. Thus, we applied co-expression cluster analyses to identify biologically relevant genes dynamically changing according to fetal weight in each fetal organ and in maternal blood. The analyses were carried out considering (i) all the fetal organs together (as a single dataset), (ii) each fetal organ dataset individually, and (iii) the three fetal organ datasets in the same analysis, to determine co-expression clusters shared between the organs.

## Materials and methods

### Animals

All experimental procedures involving animals were sanctioned by the Animal Research Ethics Committee of University College Dublin and were licensed by the Health Products Regulatory Authority, Ireland, in accordance with Statutory Instrument No. 543 of 2012 under Directive 2010/63/EU on the Protection of Animals used for Scientific Purposes.

Details of the experimental design can be found in our previous publication [19]. Briefly, heifers were randomly assigned to one of the two groups: those receiving a Day 7 embryo on Day 7 of the cycle (synchronous; ET_D7, *n* = 23) and those transferred a Day 7 embryo on Day 9 of the cycle (asynchronous; ET_D9, *n* = 33). The synchronization protocol was started 2 days earlier for heifers in the ET_D9 group, such that ET was done on the same day for both groups. Blood samples were collected in Tempus Tubes (Thermo Fisher Scientific, Waltham, MA, USA) from pregnant heifers (*n* = 25) on Day 42 of gestation, following which they were slaughtered in a commercial abattoir. The reproductive tract was recovered and held on ice until processing for sample collection, which was done within 30 min of slaughter. The pregnant uterine horn was opened along the major curvature to expose and retrieve the fetal membranes. Once the fetus was isolated, the liver, heart, and gonads were dissected, placed in individual RNAse-free tubes, and snap-frozen in liquid nitrogen. Blood and fetal organ samples were stored at −80°C. Samples corresponding to 10 male fetuses with divergent weight (equally derived from heifers in the ET_D7 and ET_D9 groups) were employed in the current study.

### RNA extraction, library preparation, and sequencing

Blood and tissues samples were shipped on dry ice to Macrogen Europe (Amsterdam, Netherlands), which performed the RNA extraction, quality control, library preparation, and sequencing. One sample from the heart, one from the gonads, and two from the liver were discarded after quality control because of the rRNA ratio and RIN <7. For the rest of the samples, libraries were prepared with Truseq-stranded mRNA for all tissue samples and SMARTer Stranded RNA library for the blood samples, followed by PE100 sequencing with the NovaSeq platform, generating ~30 million raw reads per sample in FASQ format.

### Bioinformatic analysis

The read qualities for each fastq file were accessed with FastQC (http://www.bioinformatics.babraham.ac.uk/projects/fastqc/), and low-quality bases and adapters were removed with Trimmomatic (V 0.39) [28]. The sequenced reads were mapped to the bovine reference genome (bosTau 9) with the STAR aligner (V 2.7.0b) [29], generating the genome index with the ARS-UCD1.3 assembly. On average, 86.7% of the reads were uniquely mapped to the genome, ranging from 70.6% to 93.2%. Read counts were estimated at the gene level, and the counting was done with featureCounts [30], part of the Subread software (V 2.0.3).

### Identification of clusters of co-expressed genes in the processed data

The analyses were carried out considering the following:

- All the fetal organs together (as a single dataset): Processed data from the heart, gonads, and liver were merged to generate a single table. Transcripts with zero expression in all the samples were filtered out. The resulting dataset comprised 15 746 transcripts and 26 samples (9 from the heart, 9 from gonads, and 8 from the liver). The dataset was subject to a co-expression analysis using the Clust method [31] for Python. Briefly, the method pipeline consists of four steps: data pre-processing, production of seed clusters, cluster evaluation and selection of elite seed clusters, and optimization and completion of the elite seed clusters to generate the final clusters. Details about each step can be found in the corresponding reference.
- Each fetal organ and maternal blood dataset individually: First, genes differing with fetal weight were identified by fitting a negative binomial generalized additive model to infer smooth functions for the gene expression measures along fetal weight [32], retaining genes for which the smoother coefficients were different from zero (*p* < 0.05). The number of genes selected for each dataset was 6816 (heart), 8391 (gonads), 4215 (liver), and 4938 (maternal blood). Next, these genes were next subject to co-expression cluster analysis with Clust, as explained previously.
- The three fetal organ datasets in the same analysis: The three datasets from the fetal organs were processed together to determine co-expression clusters that were correlated between the organs with the Clust software. Transcripts with zero expression in all the samples for each dataset were filtered out.

For all the analyses, genes in the co-expression clusters were functionally annotated by determining the overrepresented Gene Ontology and Uniprot Keywords biological processes and Kyoto Encyclopedia of Genes and Genomes (KEGG) pathways (false discovery rate [FDR] < 0.05) with the DAVID database [33]. Only the clusters containing genes enriching ontological terms are reported in the present study.

### Identification of genes in the maternal blood transcriptome predictive of fetal weight

The clusters of co-expressed genes determined for each fetal organ individually were compared with the cluster of co-expressed genes in the maternal blood in Venn diagrams. Statistical comparisons were made by the test of independence (Pearson’s chi-square test) to determine relatedness between the overlapping co-expressed genes in each fetal organ and the maternal blood. The expressions of these overlapping genes in each of the fetal organs were used to train a predictive regression model by extreme gradient boosting (XGboost), implemented with the caret package for R [34], tuning the parameters for the number of rounds, maximum depth, and eta, and keeping the other parameters as default. A 5-fold cross-validation was employed as the internal control for the training dataset. The testing dataset consisted of the expression of the same genes in the maternal blood. An add-on batch effect adjustment of the testing data with the training data was performed with the bapred package [35]. The quality of the fetal weight prediction was measured through the root-mean-square error (RMSE).

The selected overlapping genes were further explored through the GeneMania plugin [36] for the Cytoscape software V 3.9.1 [37]. Interactions between the genes were inferred according to co-expression, physical and genetic interactions, if they shared protein domains or participated in the same pathway. Attributes for those genes, retrieved from gene-set enrichment datasets, were also visualized.

## Results

### Genes strongly co-expressed in one fetal organ enrich ontological terms specific to that organ

**Figure 1:**
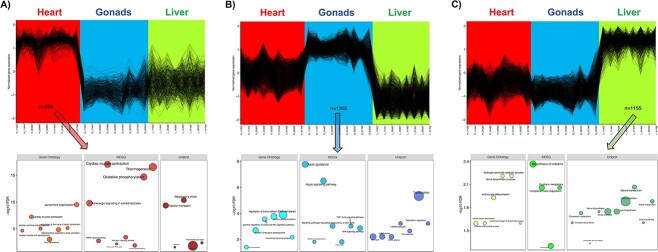
Clusters of co-expressed genes considering all fetal organs as a unique dataset. Genes in the identified clusters exhibited higher expression in (A) heart, (B) gonads, and (C) liver than in the other two organs, respectively. Below each cluster are depicted the main biologically relevant ontological terms enriched (FDR < 0.05) with the corresponding genes in each cluster. The size of the bubble corresponds to the number of genes involved in the term. Gene Ontology: Gene ontology biological process; KEGG: Kyoto Encyclopaedia of Genes and Genomes pathways; Uniprot: Uniprot keywords biological processes.

Three patterns of co-expressed genes were identified when samples from the three fetal organs were analyzed as a single dataset. Each pattern showed higher expression in the samples from one organ than in the samples from the other two organs (Figure 1, Table S1). The cluster of 524 genes with higher expression in the heart than gonads and liver enriched organ-specific terms such as cardiac muscle morphogenesis and cardiac muscle contraction, in addition to ontological terms related to mitochondrial energy production, such as oxidative phosphorylation and respiratory chain (Figure 1A). The cluster of 1305 genes with higher expression in the gonads enriched differentiation-related ontological terms, including Hippo, transforming growth factor beta (TGFB), and Wnt signaling pathway, and terms involved in protein synthesis, such as transcription and translation (Figure 1B). Finally, the 1155 genes in the up-regulated cluster in the liver were associated with erythropoiesis and lipid and cholesterol metabolism (Figure 1C). All the enriched ontological terms are listed in Table S2.

### Each fetal organ reveals clusters of co-expressed genes changing in expression according to fetal weight

**Figure 2:**
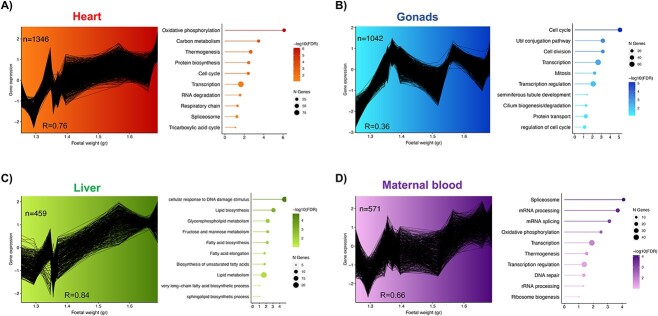
Clusters of co-expressed genes considering each fetal organ as a unique dataset. Genes in the identified clusters showed a positive correlation with fetal weight according to their expression in (A) heart, (B) gonads, (C) liver, and (D) maternal blood. The plots show the number of genes involved in the cluster and the correlation coefficient, or *R*, for gene expression and fetal weight. Next to each cluster are depicted the main biologically relevant ontological terms enriched (FDR < 0.1) with the corresponding genes on each cluster. The size of the circle corresponds to the number of genes involved in the term.

Fetal weight ranged from 1.25 to 1.69 g (mean 1.44 ± 0.15 g). For each dataset from the fetal organs and maternal blood, genes of interest were identified first as those varying with fetal weight and then filtered by co-expression to retain clusters of genes behaving similarly with increasing fetal weight. Only the clusters with enriched ontological terms are shown here. These clusters were those following a positive trend or increasing gene expression with fetal weight for all fetal organs and maternal blood (Figure 2, Table S3).

For the heart, the 1346 co-expressed genes, positively associated with fetal weight (*R* = 0.76), were involved in energy generation terms and protein synthesis (Figure 2A). For the gonads, the 1042 co-expressed genes showed an increase between 1.25 and 1.35 g, and then their expression fluctuated, likely due to individual variations. Nonetheless, these genes were more expressed in the heaviest fetus than the lightest (*R* = 0.36) and were involved in seminiferous tubule development and terms related to the cellular biology, such as cell cycle and mitosis (Figure 2B). The 459 co-expressed genes identified in the liver were positively correlated with fetal weight (*R* = 0.84) and were associated with lipid synthesis and metabolism (Figure 2C). Finally, the cluster of 571 co-expressed genes determined in maternal blood also exhibited increased expression as fetal weight increased (*R* = 0.66), and enriched some terms similarly to the fetal heart, such as oxidative phosphorylation and thermogenesis and terms related to mRNA processing. All the enriched ontological terms are listed in Table S4.

### A unique cluster of co-expressed genes was positively associated with fetal weight in each of the three fetal organs

**Figure 3:**
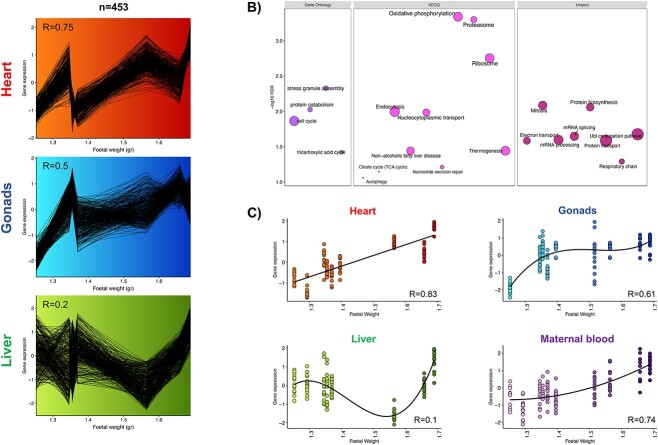
Clusters of co-expressed genes considering the three fetal organ datasets in the same analysis. (A) The identified cluster contained 454 genes whose expression positively correlated with fetal weight in the three fetal organs. (B) Ontological terms enriched (FDR < 0.05) with the 454 genes. The size of the bubble corresponds to the number of genes involved in the term. (C) Expression of genes enriching oxidative phosphorylation against fetal weight for each organ and maternal blood. The best pattern fitting the gene expression along fetal weight was determined according to the Akaike information criterion. All plots show the Pearson’s correlation coefficient, or *R*, for gene expression and fetal weight. Gene Ontology: Gene ontology biological process; KEGG: Kyoto Encyclopaedia of Genes and Genomes pathways; Uniprot: Uniprot keywords biological processes.

Next, co-expression clustering analysis was done by processing together the three datasets from the fetal organs to identify correlated clusters of genes shared between the organs. From the three resulting clusters, only the 454 genes in the cluster following a positive association between gene expression and fetal weight in each organ (Figure 3A) enriched ontological terms. These terms were mainly involved in energy generation and protein synthesis (Figure 3B, Table S5). As oxidative phosphorylation was the biologically relevant top enriched term, the expression of the 15 genes involved in this pathway in each organ was plotted against fetal weight. The same plot was done for maternal blood but using the expression of the 13 genes enriching oxidative phosphorylation out of the 571 co-expressed genes (see above). According to the Akaike information criterion, the best pattern fitting the gene expression along fetal weight (*p* < 0.001) consisted of a cubic relationship for the liver and gonads and quadratic and linear relationships for maternal blood and heart, respectively (Figure 3C). These two organs also showed the highest Pearson correlation between fetal weight and gene expression, followed by the gonad (*p* < 0.001). The Pearson correlation was not significant for liver (*p* = 0.2).

### The molecular profile of the overlapping co-expressed genes between the fetal heart and maternal blood can be modeled to predict fetal weight

**Figure 4:**
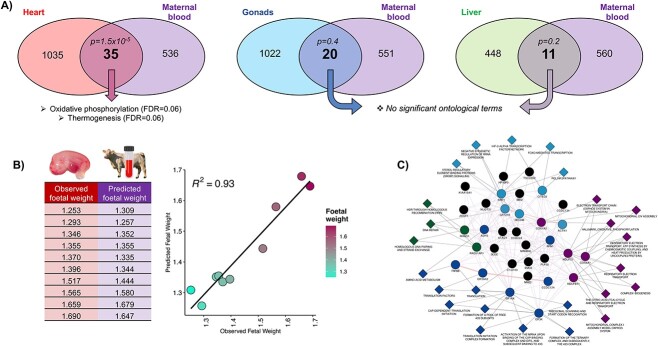
Identification of maternal blood genes predicting fetal weight. (A) The clusters of co-expressed genes for each fetal organ and maternal blood (see Figure 2) were overlapped to identify significantly associated genes between both datasets according to a chi-square test. (B) Prediction of fetal weight according to the expression of the 35 overlapped genes in maternal blood after training the model with the expression in fetal heart and corresponding regression model between the observed and predicted values. (C) Network inferred with the 35 genes and their biological attributes.

Identification of a maternal blood transcriptomic profile predictive of fetal weight was made as follows: first, the clusters of co-expressed genes determined for each fetal organ individually were compared with the cluster of co-expressed genes in the maternal blood in Venn diagrams (Figure 4A). A chi-square test yielded a significant overlap for the 35 co-expressed genes shared between the clusters identified in the fetal heart and maternal blood but not for the 20 or 11 overlapping genes between fetal gonads or liver, respectively, and maternal blood. Indeed, only the 35 overlapping genes between fetal heart and maternal blood enriched ontological terms (oxidative phosphorylation and thermogenesis). Distribution of fetal heart, or fetal liver, and maternal blood samples according to the normalized expression of the overlapping genes showed a better separation between samples belonging to heavier or lighter fetuses than for fetal gonads and blood samples (Figure S1). Next, the expression patterns of the overlapping genes in the fetal organ were employed to train a model for predicting fetal weight. The expression patterns of the same genes but in the maternal blood were used to test the model by predicting fetal weight. The highest RMSE for the prediction was when the model was trained with the 20 overlapping genes between gonads and blood (RMSE = 0.17). The RMSE was lower for the model trained with the 11 overlapping genes between the liver and blood (RMSE = 0.13). But, remarkably, the RMSE was the lowest when the model was trained with the 35 overlapping genes between heart and maternal blood (RSME = 0.04). The *R*^2^ for the regression model was 0.93 (Figure 3B), indicating that ~93% of the variance in expression of the overlapping genes in the maternal blood is explained by the variance of these genes in the fetal heart.

Exploration of the 35 genes through a network analysis demonstrated that attributes linked to several of them are related to oxidative phosphorylation, or OXPHOS complexes (mitochondrial respiration), translation initiation, DNA repair, and Hypoxia-Inducible Factor 2 Alpha/FOXO transcription factors (Figure 3C). The 35 genes are listed in Table 1.

**Table 1:** Co-expressed genes shared between the fetal heart and maternal blood positively associated with fetal weight

EntrezID	Symbol	Gene name
541277	*TSC22D2*	TSC22 domain family member 2
788792	*FARSB*	phenylalanyl-tRNA synthetase subunit beta
510194	*HP1BP3*	heterochromatin protein 1 binding protein 3
538459	*C3H1orf43*	chromosome 3 C1orf43 homolog
101903959	*LUZP6*	leucine zipper protein 6
615937	*RAD51AP1*	RAD51 associated protein 1
513642	*RHNO1*	RAD9-HUS1-RAD1 interacting nuclear orphan 1
516012	*CCDC134*	coiled-coil domain containing 134
532862	*SEC24B*	SEC24 homolog B, COPII coat complex component
505515	*ADH5*	alcohol dehydrogenase 5 (class III), chi polypeptide
510778	*CCDC124*	coiled-coil domain containing 124
327688	*COX7A2*	cytochrome c oxidase subunit 7A2
613606	*AKAP7*	A-kinase anchoring protein 7
521378	*CITED2*	Cbp/p300 interacting transactivator with Glu/Asp rich carboxy-terminal domain 2
524770	*ACTN1*	actinin alpha 1
347700	*EIF2AK2*	eukaryotic translation initiation factor 2 alpha kinase 2
526913	*PRORSD1*	prdX-deacylase domain-containing protein 1
538151	*KIAA1841*	KIAA1841 ortholog
787482	*MANBAL*	mannosidase beta like
512824	*PUF60*	poly(U) binding splicing factor 60, transcript variant 1
504257	*SCOC*	short coiled-coil protein
282289	*NDUFC1*	NADH:ubiquinone oxidoreductase subunit C1
282199	*COX6A1*	cytochrome c oxidase subunit VIa polypeptide 1
533611	*BBS2*	Bardet-Biedl syndrome 2
518859	*NUDT21*	nudix hydrolase 21
515326	*EIF3K*	eukaryotic translation initiation factor 3 subunit K
613551	*EMC6*	ER membrane protein complex subunit 6
615447	*NME2*	non-metastatic cells 2, protein (NM23B) expressed in
513649	*CCDC43*	coiled-coil domain containing 43
507939	*RPN1*	ribophorin I
506045	*ATAD1*	ATPase family, AAA domain containing 1
613629	*SIRT1*	sirtuin 1
513510	*GTF2H1*	general transcription factor IIH subunit 1
404161	*NDUFB11*	NADH:ubiquinone oxidoreductase subunit B11
511189	*EIF1AX*	eukaryotic translation initiation factor 1A X-linked

## Discussion

The results of the present study demonstrate that differences in fetal weight are associated with molecular changes in key organs such as the heart, gonads, and liver and, strikingly, in the maternal blood transcriptome. Differential fetal growth was induced using an asynchronous embryo transfer model, which exposed the uterus to higher P4 concentrations in the first week post-ovulation, influencing the endometrial environment and impacting conceptus and fetal development [19]. We studied only male fetuses to account for the commencement of testes differentiation after the peak in sex-determining region Y *(SRY)* expression [38], although future similar studies can be done in female fetuses. We first performed an exploratory analysis by considering all the fetal organs as a unique dataset, which yielded clusters of co-expressed genes more expressed in one of the three organs and related to organ biology. These results indicate that in the bovine, the first of the two main transcriptional shifts during development occurs at a similar gestational age than in humans [22], the second one occurring around or after birth. We followed next with an individual analysis for each organ, identifying co-expressed genes increasing in expression with fetal weight and entailing the organ maturation.

For the heart, the co-expressed genes were involved not only in cardiac muscle morphogenesis and contraction but also in energy generation through oxidative phosphorylation. The heart originates from the mesoderm and is the first organ to differentiate in gestation; a protuberance is already observed at 22 days of gestation in the bovine embryo [20]. In mice, mitochondria found in cardiomyocytes are immature during the embryonic period, and the cardiomyocytes acquire ATP through anaerobic glycolysis [39]. However, during the transition between the embryonic and fetal stages, the OXPHOS complexes assemble to initiate the electron transport chain and ATP generation through mitochondrial respiration [40]. This metabolic shift toward aerobic oxidative phosphorylation in cardiac mitochondria is stimulated by an adrenergic signaling [41], one of the enriched KEGG pathways for the gene cluster with higher expression in the heart. Thus, our results suggest that oxidative phosphorylation is already occurring in the 42-day fetal heart, and accordingly, it is increased in heavier than lighter fetuses of the same gestational age.

For the gonads (testicles), several of the enriched terms were related to pathways involved in organ development, such as the Hippo signaling [42] and the TGFB and Bone Morphogenetic Protein signaling pathways, both of which control fetal testis development [43]. In the bovine fetus, differentiation of the male bovine gonad occurs at days 41 to 42 of gestation, after a peak in the expression of the SRY protein on day 39, followed by a decrease in expression [44]. The principal target of the SRY protein is the SRY-box 9 *(SOX9*)**, in addition to other genes that differentiate the gonad into a testis. Expression of *SOX9* orchestrates testicular morphogenesis, as it induces the differentiation of the fetal Sertoli cells in mouse [45], although in cattle, *SOX10*, but not *SOX9*, increased in expression from Days 35 to 55 of gestation [44]. We found that *SOX9* was among the co-expressed genes increasing in expression with fetal weight, which also enriched seminiferous tube development, suggesting that heavier fetuses had more differentiated gonads at least at 42 days of gestation.

The main ontological terms enriched with the cluster of genes more expressed in the liver than heart and gonads were related to erythrocyte differentiation and lipid metabolism. The liver is a hematopoietic organ during fetal life and the site of development of adult-type red blood cells [46]. In the human fetus, the hematopoietic stem cells colonize the liver at 23 and 30 days of gestation to expand in this organ and seed the bone marrow by 10.5 weeks [47]. Results from the present study indicate that hematopoiesis is already occurring in the liver of the 42-day bovine fetus. Another important hepatic role is fatty acid and cholesterol synthesis. Human fetuses obtain these lipids from the maternal circulation by transplacental transfer until the fatty acid and cholesterol synthesis de novo in fetal adipose tissue and liver prevails [48]. In rats, the fetal liver synthesizes fatty acid [49] and cholesterol [50] at a higher rate than in adults. In ruminants, some fatty acids can cross the placenta [51], but their main origin during fetal development is unclear. Our results suggest that the fetal liver is a source of fatty acids for the bovine fetus as early as 42 days of gestation and that this activity increases in line with fetal growth.

Another remarkable finding from this study was the identification of co-expressed genes in the maternal blood increasing in expression with fetal weight, which were involved in oxidative phosphorylation and mRNA processing. These ontological terms were also enriched by the co-expressed genes increasing in expression in all the organs. Oxidative phosphorylation is the main metabolic pathway for energy production of healthy cells, as it yields more than 30 molecules of ATP per glucose molecule, in contrast to 2 molecules of ATP by anaerobic glycolysis [52]. The molecular profile of each organ indicated increased maturation in concordance with fetal weight, which would demand higher cellular energy generation. Indeed, a study consisting of a cross-organ transcriptomic comparison for mouse brain, heart, kidney, and liver demonstrated that genes increasing in expression with organ maturation enriched terms such as oxidative phosphorylation, electron transport chain, and mitochondrial matrix [53]. The heart is the first organ to form [54], and in cattle as in humans, it is likely more developed than the liver and gonads by Day 42 [22]. Thus, the correlation between the expression of cardiac genes involved in oxidative phosphorylation and fetal weight was the highest. Notably, the correlation between maternal blood gene expression (part of this metabolic pathway) and fetal weight was the second highest. Indeed, there was an association between co-expressed genes in maternal blood and fetal heart positively increasing with fetal weight, given the overlap of 35 co-expressed genes. Furthermore, the model constructed with the 35 cardiac genes was successful in predicting fetal weight from their expression in maternal blood with low error, even when the differences in weight between the same-age-fetuses are relatively subtle (in the magnitude of milligrams). Maternal blood acting as a sensor of fetal development might be attributed to the interlink in development between the fetal heart and placenta [24, 55], which in turn can signal maternal blood, as observed in humans [1]. In addition, peripheral blood mononuclear cells from pregnant women are more likely to utilize oxidative phosphorylation to cover their energy demands than glycolysis, as in non-pregnant women [56]. Of course, a direct interpretation of results found in women to explain this phenomenon in ruminants should be made cautiously, given the differences in placentation between these species.

Estimating normal pregnancy progression and diagnosing pregnancy pathologies through maternal blood molecular profile is a hot topic in humans [57, 58]. In animals, however, this area is scarcely explored. The maternal metabolome can be employed in mares to predict gestational age [59]. In cattle, circulating microRNAs in the maternal plasma differ according to the fetal sex at 39 days of gestation [60], and oversized fetuses induce changes in the maternal blood transcriptome at 55 and 105 days of gestation [61]. Here, we linked the information from the fetal heart and maternal blood to accurately predict fetal weight in 42-day fetuses. Our results can shed light on the biological underpinnings of maternal–fetal development in ruminants and potentially help to develop non-invasive tools for fetal wellbeing assessment.

## Supplementary Material

TableS1_ioad103

TableS2_ioad103

TableS3_ioad103

TableS4_ioad103

TableS5_ioad103

FigureS1_ioad103

## Data Availability

Data are deposited in NCBI’s Gene Expression Omnibus and are accessible through GEO accession number GSE230294 (https://www.ncbi.nlm.nih.gov/geo/query/acc.cgi?&acc=GSE230294).
