# Supplementary figures and images for: Maternal blood transcriptome as a sensor of fetal organ maturation at the end of organogenesis in cattle
Source: Biol Reprod. 2023 Sep 2;109(5):749–58. doi: 10.1093/biolre/ioad103 (PMC10651065; doi:10.1093/biolre/ioad103)

Heart / Blood - 35 genes

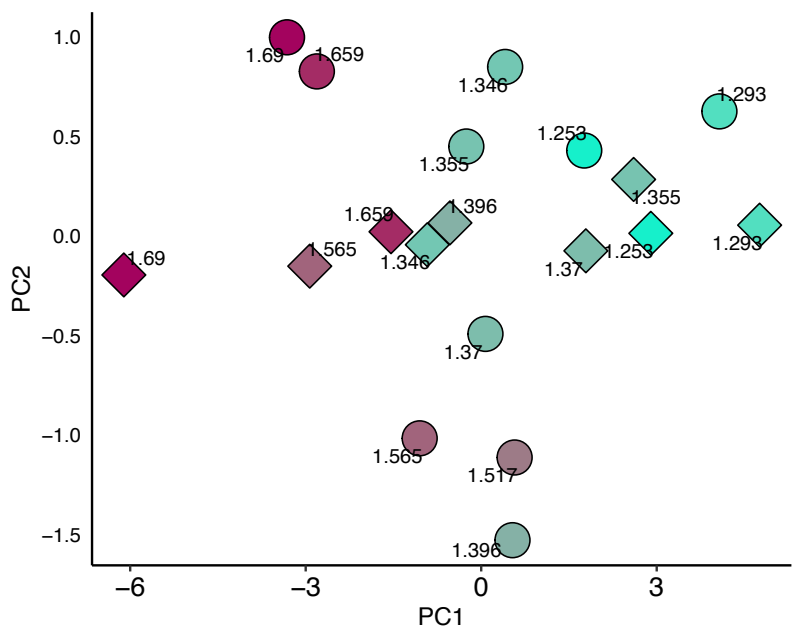

Gonad / Blood - 20 genes

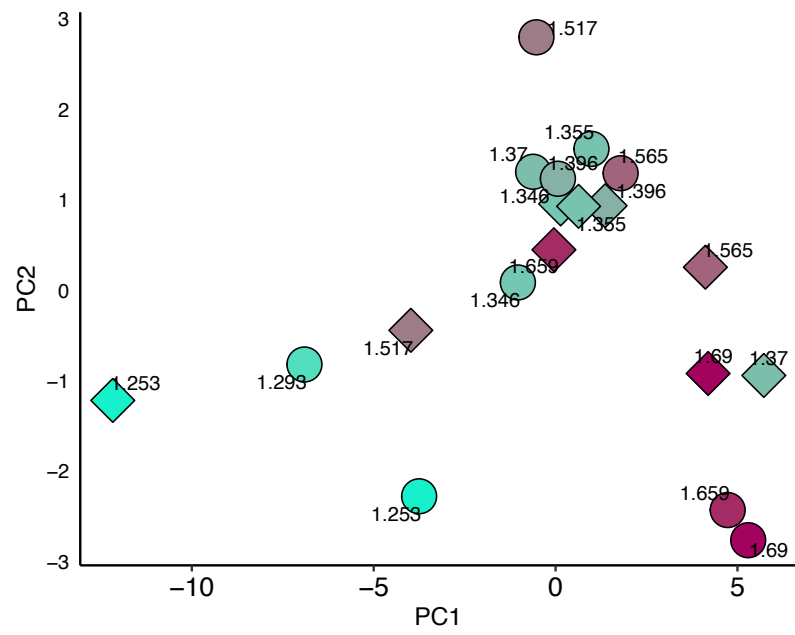

Liver / Blood - 11 genes

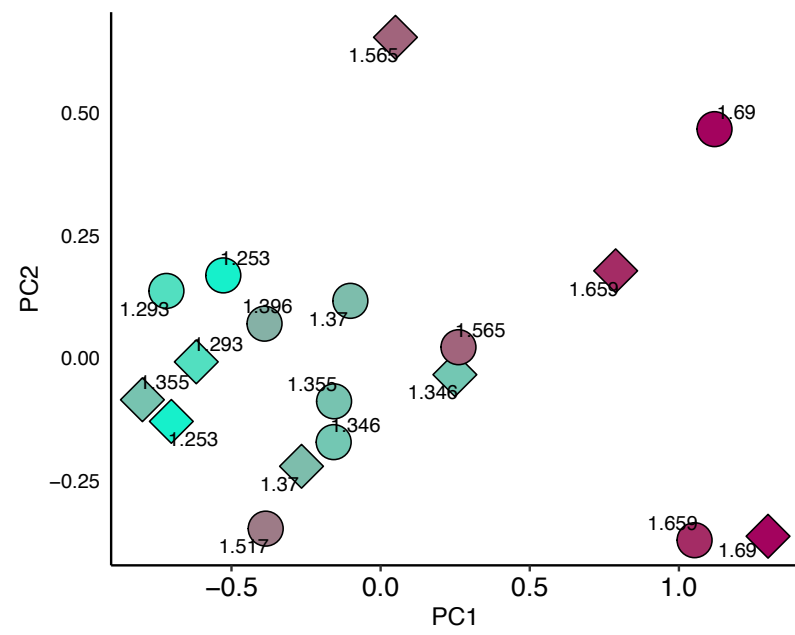

Maternal Blood      Foetal Organ

Supplement: FigureS1_ioad103 [file figures1_ioad103.pdf]
